# Study of the Mechanical Properties and Microstructure of Alkali-Activated Fly Ash–Slag Composite Cementitious Materials

**DOI:** 10.3390/polym15081903

**Published:** 2023-04-15

**Authors:** Yigang Lv, Cui Wang, Weiwei Han, Xing Li, Hui Peng

**Affiliations:** 1 Key Laboratory of Advanced Engineering Materials, Structure Behavior and Functional Control of University of Hunan Province, Changsha University of Science & Technology, Changsha 410114, China; 2 School of Civil Engineering, Changsha University of Science & Technology, Changsha 410114, China; 3 School of Traffic & Transportation Engineering, Changsha University of Science & Technology, Changsha 410114, China

**Keywords:** alkali activated, fly ash–slag, composite cementitious materials, compressive strength, microstructure

## Abstract

Composites that use fly ash and slag as alkali-activated materials instead of cement can overcome the defects and negative effects of alkali-activated cementitious materials prepared with the use of an alkali-activated material. In this study, fly ash and slag were used as raw materials to prepare alkali-activated composite cementitious materials. Experimental studies were carried out on the effects of the slag content, activator concentration and curing age on the compressive strength of the composite cementitious materials. The microstructure was characterized using hydration heat, X-ray diffraction (XRD), Fourier transform infrared spectroscopy (FT-IR), mercury intrusion porosimetry (MIP) and scanning electron microscopy (SEM), and its intrinsic influence mechanism was revealed. The results show that increasing the curing age improves the degree of polymerization reaction and the composite reaches 77~86% of its 7-day compressive strength after 3 days. Except for the composites with 10% and 30% slag content, which reach 33% and 64%, respectively, of their 28-day compressive strength at 7 days, the remaining composites reach more than 95%. This result indicates that the alkali-activated fly ash–slag composite cementitious material has a rapid hydration reaction in the early stage and a slow hydration reaction in the later stage. The amount of slag is the main influencing factor of the compressive strength of alkali-activated cementitious materials. The compressive strength shows a trend of continuous increase when increasing slag content from 10% to 90%, and the maximum compressive strength reaches 80.26 MPa. The increase in the slag content introduces more Ca^2+^ into the system, which increases the hydration reaction rate, promotes the formation of more hydration products, refines the pore size distribution of the structure, reduces the porosity, and forms a denser microstructure. Therefore, it improves the mechanical properties of the cementitious material. The compressive strength shows a trend of first increasing and then decreasing when the activator concentration increases from 0.20 to 0.40, and the maximum compressive strength is 61.68 MPa (obtained at 0.30). The increase in the activator concentration improves the alkaline environment of the solution, optimizes the level of the hydration reaction, promotes the formation of more hydration products, and makes the microstructure denser. However, an activator concentration that is too large or too small hinders the hydration reaction and affects the strength development of the cementitious material.

## 1. Introduction

Traditional Portland cement is the most widely used building material in China, but a large amount of CO_2_ is generated during the production process, causing a series of problems related to environmental pollution. Therefore, it is necessary to identify a low-carbon green cement as part of a new generation of building materials in order to ensure the sustainable development of civil engineering in China. Alkali-activated materials are natural mineral or industrial waste materials that are rich in silicon-aluminum activated by an alkaline solution; inorganic gel materials with a spatial network structure are generated after the dissolution and polymerization of ionic bonds and covalent bonds. These materials are characterized by their light weight, initial strength, and lower levels of carbon emissions generated during the preparation process than in the process of making traditional Portland cement [1,2,3,4,5,6,7,8,9,10,11].

The most commonly used alkali-activated materials are rich in silicon-aluminum industrial by-products, or minerals such as fly ash, slag and metakaolin. Alkali-activated fly ash has excellent properties, such as high strength, low shrinkage, high temperature resistance and good stability. However, due to the low activity of fly ash, as well as its slow reaction curing, it needs to be cured under high temperatures, high levels of humidity, and other environmental conditions, and it has poor anti-permeability. Moreover, the composition of different types of fly ash varies, so the performance of the prepared alkali-activated materials varies greatly, which limits the actual use of alkali-activated fly ash materials in engineering [12,13,14,15,16,17]. Nath et al. [18] and Ling et al. [19] found that the incorporation of a certain amount of slag in an alkali-activated fly ash system can effectively improve the defects that exist in single use conditions. The incorporation of slag can significantly accelerate its hydration rate, promote the formation of a large number of C-S-H, C-A-S-H, and N-A-S-H gels, and form a denser microstructure. The increase in slag content introduces more Ca+ into the reaction system, which promotes a polymerization reaction and is conducive to the improvement of the material’s mechanical properties and microstructure compactness [20,21,22]. However, when using slag to prepare alkali-activated materials, the main hydration product is a C-S-H gel with a low calcium–silicon ratio, which has the advantages of high initial strength, low porosity, and excellent anti-permeability. However, it also has the disadvantages of a poor working performance, short setting time, and large shrinkage of the hardened body [23,24,25]. Shi et al. [26] found that the incorporation of an appropriate amount of fly ash can improve the fluidity of alkali-activated slag and prolong the coagulation time. At present, scholars in China and abroad have found that the slag content, water–binder ratio, concentration, and modulus of the alkaline activator, as well as the curing conditions, all have a certain impact on the performance of alkali-activated cementitious materials [27,28,29]. Lee et al. [30] found that, with increased slag content, the compressive strength of the alkali-activated fly ash–slag was higher; the strength was lowered with an increase in the water–binder ratio. Elyamany et al. [31] evaluated the mechanical properties and microstructure of geopolymer mortar. It was found that increasing the curing temperature and the molar concentration of the sodium hydroxide solution could effectively improve its compressive strength and flexural strength. Regarding alkali-activated slag, some scholars suggest that its compressive strength increases with the increase in the modulus [32], but others contend that the increase in the modulus reduces the compressive strength of alkali-activated slag [33]. Yang et al. [34] used XRD, FT-IR, and SEM to analyze the microstructure of alkali-activated cementitious materials, and found that the increase in fly ash reduced the formation of tobermorite, inhibited the crystallization of calcium silicate hydrate, and reduced the strength of the material. The increase in the activator concentration increased the degree of fly ash activation. The combined use of fly ash and slag allows the materials to display their respective advantages, while each benefits from the other’s strengths, overcoming the shortcomings that arise when using a single alkali-activated material.

Alkali-activated materials often have problems when used alone, but can produce a superposition effect when used together. Alkali-activated materials with different characteristics can play a role in different periods of the hydration and structure formation process, so as to achieve the effect of complementing each other. In order to achieve the best gel cementation effect in the mixture of various raw materials, it is necessary to find the most suitable raw material proportion and activator concentration. At present, there is a lack of research on the appropriate activator concentration in the alkali-activated complex system, and there is a lack of in-depth analysis of the microscopic mechanism. The relationship between the proportion of raw materials, the concentration of activator and the properties of gel, the evolution law and the internal influence mechanism have not been clearly revealed. In this paper, fly ash and slag are used as alkali-activated materials to prepare alkali-activated fly ash–slag composite cementitious materials. The effects of the slag content and activator concentration on the compressive strength of alkali-activated fly ash–slag composite cementitious materials at different curing ages are studied, and the optimal slag content and activator concentration are found. The hydration reaction rate, phase composition, pore distribution characteristics, and micromorphology of alkali-activated cementitious materials are characterized by hydration heat, XRD, FT-IR, MIP, and SEM, and other technical means. Moreover, we explore the intrinsic influence mechanism of the slag content and activator concentration on the material’s mechanical properties, in order to provide guidance for the study of other properties of alkali-activated cementitious materials, and to provide a theoretical basis for application in practical engineering.

## 2. Experimental Design

### 2.1. Materials

**Figure 1:**
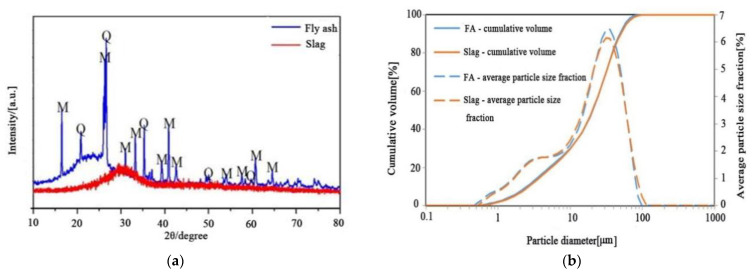
(**a**) XRD diagram of the materials (M-mullite; Q-quartz); (**b**) particle size distribution of the materials.

The test uses grade I fly ash and S95 grade blast-furnace slag; specific chemical components were obtained by XRF analysis, as shown in Table 1. The chemical composition of fly ash mainly comprises SiO_2_ and Al_2_O_3_, and the main components of slag are CaO, SiO_2_, and Al_2_O_3_. An X-ray diffraction analysis of the fly ash and slag is shown in Figure 1a, from which it can be seen that the slag is characterized by an amorphous phase; there are two crystalline phases in fly ash, namely mullite and quartz. Figure 1b shows the particle size distribution of slag and fly ash. This is mainly distributed in the range of 0.5–100 μm, and the median particle sizes are 20.19 μm and 20.46 μm, respectively.

The alkaline activator is a mixed solution of liquid sodium silicate, solid sodium hydroxide, and water. Sodium silicate adopts the weakly alkaline liquid sodium silicate produced by Neiqiu Litian Chemical Co., Ltd., Xingtai City, Hebei Province, in which the mass fractions of Na_2_O and SiO_2_ are 8.35% and 26.54%, respectively, and the modulus is 3.28. Solid sodium hydroxide is an industrial-grade flake sodium hydroxide produced by Zhengzhou Qingyuan Chemical Products Co., Ltd., Henan Province, with a purity of 99.5%.

### 2.2. Specimen Preparation and Mixed-Ratio Design

In this study, fly ash and slag are used as alkali-activated materials, and the mixed solution of liquid sodium silicate, solid sodium hydroxide and water is used as the alkaline activator to prepare alkali-activated fly ash–slag composite cementitious materials. The water-to-binder ratio is defined as the ratio of the sum of the masses of the external water and the water in the sodium silicate to the sum of the masses of the raw material and the activator (excluding the water in the sodium silicate). Modulus is defined as the molar ratio of SiO_2_ and Na_2_O in the activator. The water-binder ratio and modulus of all samples are 0.35 and 1.2, respectively. With the mass proportion of slag in raw materials and the activator concentration (the ratio of the sum of the masses of SiO_2_, Na_2_O in sodium silicate and solid sodium hydroxide to the mass of the activator solution.) as research variables, a total of 10 sets of mixed-ratio schemes were designed, as shown in Table 2. The alkaline activator was prepared according to the target values of the water–binder ratio, the activator modulus, and the activator concentration of the experimental design. The weighed solid sodium hydroxide was added to the liquid sodium silicate; finally, water was added, placed in a closed container, and stirred for 10 h with a magnetic stirrer to obtain a modified sodium silicate solution. According to the design of the mixing ratio, the configured alkaline activator was poured into the mixer, equipped with fly ash and slag for stirring, and the stirred slurry was poured into a 70.7 mm × 70.7 mm × 70.7 mm cube mold. After 24 h, the mold was removed and wrapped with plastic film, and the test block was placed in a standard curing box with a temperature of 20 °C and a relative humidity of 90% for 3 d, 7 d, and 28 d.

### 2.3. Test Methods

#### 2.3.1. Compressive Strength Test

The compressive strength test method was carried out in accordance with the relevant Chinese standards [35]. Three specimens were tested in each group. The specimens were cube test blocks with a size of 70.7 mm × 70.7 mm × 70.7 mm. When the specimen reached the curing age required by the design, the surface of the specimen was wiped clean, the size of the specimen was checked, and the compressive strength test of the cube was carried out. Continuous and uniform loading was carried out during the test, and the loading speed was 1.5 kN/s. The specimen was automatically unloaded after failure and the strength was recorded. 

#### 2.3.2. Reaction Heat Test 

The reaction heat test was conducted using an American Waters TAM-AIR-series microcalorimeter. Then, 1 g of each group of raw materials and the corresponding quality of alkaline activator was taken, and the micro-heat generated in the process of material dissolution, polymerization, and curing was monitored and measured using the microcalorimeter. The ambient temperature for the whole test process was 25 °C, and the monitoring time was more than 50 h.

#### 2.3.3. Phase Composition Test

The XRD test uses the Japanese Rigaku Ultima IV series X-ray diffractometer, with an XRD scanning range of 5–90° and a scanning speed of 5°/min. Part of the fragments from the middle of the sample were knocked out, the fragments put into the test tube and soaked in anhydrous ethanol to stop the hydration. The hydrated samples were then dried in a 60 °C oven and ground to powder in a grinding bowl. The powder was tested with a 200 target sieve.

The FT-IR test was conducted using the Shimadzu IRTracer-100 series Fourier-transform infrared spectroscopy analyzer in Japan, with an FT-IR scanning range of 400–4000 cm^−1^ and a resolution of 4 cm^−1^. Part of the fragments from the middle of the sample were knocked out, the fragments put into the test tube and soaked in anhydrous ethanol to stop the hydration. Then the sample with terminated hydration was dried in a 60 °C oven and ground to powder in the grinding bowl. The powder was taken through a 200 target sieve and mixed with KBr tablet for testing.

#### 2.3.4. Pore Structure Test

The MIP test was conducted using a PoreMaster 60 series automatic aperture analyzer, and the diameter measurement was carried out at 0.0036~1100 μm. Part of the fragments from the middle of the sample were knocked out, the fragments put into the test tube and soaked in anhydrous ethanol to stop the hydration. Then the hydrated sample was put into a 60 °C oven to dry, and the dried sample MIP tested.

#### 2.3.5. Micromorphology Test

The SEM test used a German Zeiss EVO MA 25 series high-resolution scanning electron microscope. Part of the fragments from the middle of the sample were knocked out, the fragments put into the test tube and soaked in anhydrous ethanol to stop the hydration, and then the terminated hydration sample was put into a 60 °C oven to dry, selecting a sample with a flat surface for gold spraying, increasing its conductivity, ensuring the quality of scanning, and finally observing the microscopic morphology of the sample.

## 3. Results and Discussion

### 3.1. Compressive Strength Analysis

**Figure 2:**
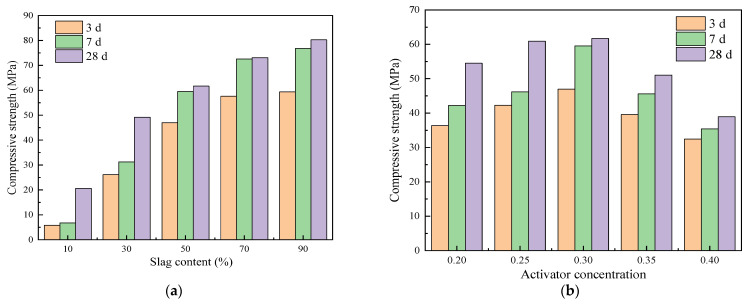
(**a**) The relationship between compressive strength and slag content at different ages; (**b**) the relationship between compressive strength and the activator concentration at different ages.

The variation in compressive strength with slag content at different curing ages is shown in Figure 2a. It can be seen from the figure that the compressive strength increases with the increase in the curing age, and the 3-day compressive strength of alkali-activated cementitious materials under different ratios reaches 77–86% of the 7-day compressive strength. The alkali-activated cementitious materials with 10% and 30% slag content reached 33% and 64%, respectively, of their 28-day compressive strength levels on day 7; the rest of the materials reached levels of more than 95%. This indicates that the strength of alkali-activated fly ash–slag composite cementitious materials develops rapidly, and its initial strength is easily demonstrated. This is consistent with the experimental research results of Huang et al. [21]. As the slag content increases from 10% to 90%, the compressive strength shows a trend of continuous increase. At the age of 28 days, the compressive strength of the material with 10% slag content is 20.61 MPa. When the slag content increases to 30%, the compressive strength increases to 49.14 MPa; compared with the compressive strength of the material with 10% slag content, the growth rate is 138%. When the slag content continues to increase to 50% and 70%, the compressive strength reaches 61.18 MPa and 73.04 MPa, respectively; compared with the compressive strength of the previous slag content levels, the growth rate is 24.50% and 19.39%, respectively. When the slag content reaches 90%, the compressive strength is 80.26 MPa; compared with the compressive strength of the material with 70% slag content, the growth rate is only 9.88%. This indicates that, as the slag content increases, the compressive strength of alkali-activated cementitious materials is effectively improved; with the continuous increase in slag content, the speed of the increase in compressive strength continues to decrease. This is because the increase of slag content promotes the hydration reaction rate of alkali-activated materials, generates more hydration products, and refines the pore size distribution of the structure. The porosity of the structure is reduced, and the compressive strength of the alkali-activated cementitious material is increased [21].

The variation in compressive strength with activator concentration at different curing ages is shown in Figure 2b. It can be seen that, as the activator concentration increases from 0.20 to 0.40, the compressive strength of alkali-activated cementitious materials first increases and then decreases; an activator concentration of 0.30 has the best effect, reaching 61.18 MPa in 28 days. When the activator concentration increases from 0.20 to 0.30, the compressive strength of the alkali-activated cementitious material at 3 days, 7 days, and 28 days increases from 36.36 MPa, 42.20 MPa, and 54.50 MPa to 46.98 MPa, 59.54 MPa, and 61.68 MPa, respectively, with the compressive strength reaching the maximum values. When the activator concentration continues to increase from 0.30 to 0.40, the compressive strength shows a downward trend. The compressive strength at 3 days, 7 days, and 28 days at 0.40 activator concentration is 32.42 MPa, 35.39 MPa, and 38.91 MPa, respectively, and the compressive strength is the lowest. At the age of 28 d, the compressive strength of 0.20, 0.25, 0.30, 0.35, and 0.40 activator concentrations are 54.50 MPa, 59.92 MPa, 61.68 MPa, 51.03 MPa, and 38.91 MPa, respectively. This indicates that, when the activator concentration is too high or too low, the development of the strength of the cementitious material is limited; meanwhile, the appropriate activator concentration can improve the compressive strength of the cementitious material. However, compared with the slag, the contribution of the activator concentration to compressive strength is small. This is because, with the increase of activator concentration, the alkaline environment of the material is improved, so that the raw material particles are dissolved more fully and the hydration rate is accelerated. More gels are formed to fill the porous system, resulting in a denser structure and increased strength [7]. Wang et al. [36] also found that the strength of cementitious materials could be improved by increasing the activator concentration. Alkali solution with high PH value could promote the dissolution rate of calcium, aluminum, silicon and other elements in the materials, and generate more hydration products, thus improving the strength of cementitious materials.

### 3.2. Reaction Rate Analysis

**Figure 3:**
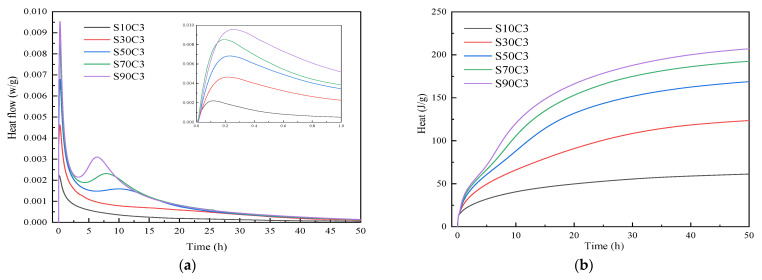
Comparison of the reaction heats of alkali-activated composite cementitious materials under different slag contents: (**a**) exothermic reaction rate; (**b**) total heat release.

**Figure 4:**
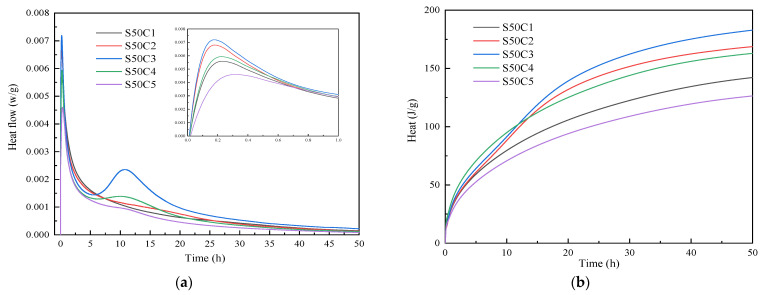
Comparison of the reaction heats of alkali-activated composite cementitious materials under different activator concentrations: (**a**) exothermic reaction rate; (**b**) total heat release.

The hydration heat test results of alkali-activated fly ash–slag composite cementitious material for different proportions are shown in Figure 3 and Figure 4. From the exothermic reaction rate curve, we can see that the hydration reaction of alkali-activated composite cementitious materials is fast, and the reaction rate is greatly slowed down after 24 h and then gradually stabilizes.

From Figure 3, it can be seen that, when the slag content is low (10% and 30%), only one exothermic peak appears in the exothermic reaction rate curve, and two exothermic peaks appear with the increase in slag content. The first is the initial exothermic peak, which reflects the dissolution process of the Si-O bond and Al-O bond breaking in the raw material. The second is the exothermic peak in the accelerated period, which reflects the process of gradual polymerization and the arrangement of monomers and gel generation [28]. The exothermic reaction rate of materials with different slag contents essentially reaches the maximum within 0.4 h; that is, the initial exothermic peak appears. This indicates that the alkali-activated cementitious material has a rapid hydration reaction and promotes the formation of a large number of reaction products, which may be the main reason for the early strength characteristics of alkali-activated cementitious materials. When the slag content is low, due to the large amount of fly ash, the hydration activity is also low. Under the action of an alkaline activator, the exothermic reaction rate appears within 0.4 h of the initial exothermic peak. However, the highest exothermic reaction rate is low, although the hydration reaction continues afterwards. Nonetheless, the reaction rate and total exothermic heat are low, the hydration reaction is very slow, and the hydration product is generated, resulting in lower strength. With the increase of slag content, the peak value of the initial exothermic peak increases continuously, which indicates that the dissolution rate of raw materials increases continuously. When the slag content is 50%, the second exothermic peak begins to appear along with the progress of the reaction, which indicates that the monomer gradually polymerizes and forms, a large number of gels are continuously generated, and the strength is constantly improved. The test data are compared and analyzed. With the increase of slag content, the exothermic reaction rate and total heat release increases continuously, the highest exothermic reaction rate increases from 0.0023 w/g to 0.0095 w/g, and the total heat release increases from 62.72 J/g to 210.93 J/g. This indicates that, in the same alkaline environment, the reactivity of slag is higher than that of fly ash. With the increase in slag content, a large amount of Ca^2+^ dissolves, resulting in a more violent reaction in the dissolution stage, which is conducive to the hydration reaction, promotes the formation of a large number of hydration products, and greatly improves the strength of alkali-activated cementitious materials [21].

From Figure 4, it can be seen that the reaction exothermic rate curves of different activator concentrations have an initial exothermic peak within 0.3 h. The second exothermic peak occurs when the activator concentration is 0.30 and 0.35 as the reaction continues. When the activator concentration increases from 0.20 to 0.30, the peak value of the initial exothermic peak increases continuously, and a second exothermic peak appears. The exothermic reaction rate and total exothermic heat show an increasing trend. Because the increase in the alkali concentration introduces more Na_2_O and SiO_2_ into the system, it increases the PH of the reaction system. The dissolution reaction between fly ash and slag is more violent, and the active ions of groups such as Ca^2+^ and [AlO_4_] released by dissolution can quickly react with the oligomer SiO_2_ in the initiator to form a gel precipitate, which improves the strength of the alkali-activated cementitious material. When the activator concentration continues to increase to 0.40, the peak value of the initial exothermic peak and accelerating exothermic peak decrease continuously. The reaction exothermic rate and total heat release show a decreasing trend. This is because the activator concentration is too high to cause the system to quickly generate a gel, and the generated gel quickly wraps the undissolved silicon-aluminum phase. Preventing the alkaline activator from reacting with the internal silicon-aluminum phase, reducing the degree of reaction in the system, and reducing the hydration products generated results with a decrease in the strength of alkali-activated cementitious materials [37]. From what has been discussed above, appropriately increasing the concentration of activator can effectively improve the hydration reaction rate of alkali-activated materials, promote the generation of more high-strength gels, and thus improve the strength of alkali-activated cementitious materials. 

### 3.3. X-ray Diffraction Analysis

**Figure 5:**
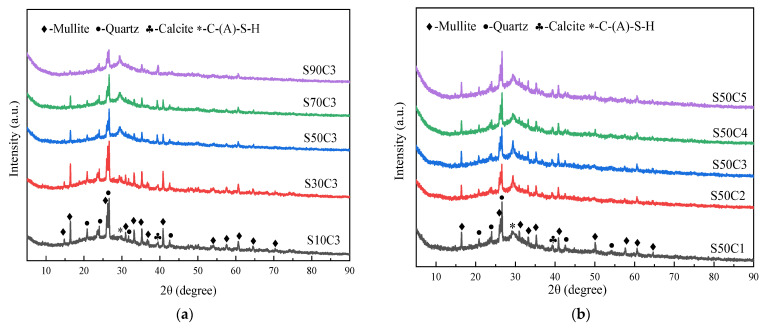
XRD pattern analysis of alkali-activated composite cementitious materials: (**a**) slag content; (**b**) activator concentration.

The XRD patterns of alkali-activated fly ash–slag composite cementitious materials at seven days of age under different slag content and activator concentrations are shown in Figure 5. It can be seen that the alkali-activated fly ash–slag composite cementitious material is mainly composed of amorphous crystal, quartz, mullite, and C-(A)-S-H gel [34]. The peak–valley range of the XRD of the cementitious material changes slightly under different proportions. This indicates that the slag content and activator concentration have a certain influence on the phase composition of the alkali-activated composite cementitious material.

Figure 5a shows the XRD diagram of alkali-activated composite cementitious material under different slag levels. When the slag content is 10%, there is a large amount of quartz and mullite in the cementitious material. This is due to the small Ca^2+^ content in the system; the hydration reaction is slow, and the hydration products are mainly N-A-S-H gels and quartz, and mullite crystal phases that are not involved in the reaction. When the slag content increases to 30%, at 2θ = 29°, C-(A)-S-H diffuse steamed-bun-like broad peaks begin to appear. Ishwarya et al. [7] found that the mineralogical composition of the alkali-activated composite system showed a wide, diffuse peak, which moved to the right in the range of 2θ = 16–39°, indicating the formation of C-S-H gel, and the area of these ridges increased with the increase of slag content in the slurry. This is consistent with our experimental phenomenon. As the slag content continues to increase, the strength of the C-(A)-S-H crystal phase peak increases, the quartz and mullite crystal phase peaks weaken, and there is also trace calcite formation. This is because, with the increase in slag content, more Ca^2+^ is introduced into the system, and the hydration reaction is accelerated. Additionally, more C-(A)-S-H gels are generated, thereby improving the strength of the cementitious material, and more Ca^2+^ reacts with CO_2_ in the air to form calcite.

From Figure 5b, it can be seen that the crystal phase peaks of quartz, mullite, calcite, and C-(A)-S-H are all present in the cementitious materials, and the relative strength changes are small. This indicates that the activator concentration has little effect on the phase composition of alkali-activated composite cementitious materials. Observing the C-(A)-S-H crystal phase peak at 2θ = 29°, it was found that, when the activator concentration increases from 0.20 to 0.30, the intensity of the crystal phase peak shows a trend of continuous increase. The activator concentration continues to increase to 0.40, and the intensity of the crystal phase peak decreases. This is because the increase in the activator concentration increases the alkaline environment of the solution, promotes the level of hydration reaction, produces more hydration products, and improves the strength of the cementitious material. However, when the activator concentration reaches a certain value, continuing to increase the concentration of activator will inhibit the hydration reaction of alkali-activated materials and reduce the strength of cementitious material [7]. In this study, the optimal concentration of activator of alkali-activated composite cementitious materials is 0.30.

### 3.4. Fourier Infrared Spectroscopy Analysis

**Figure 6:**
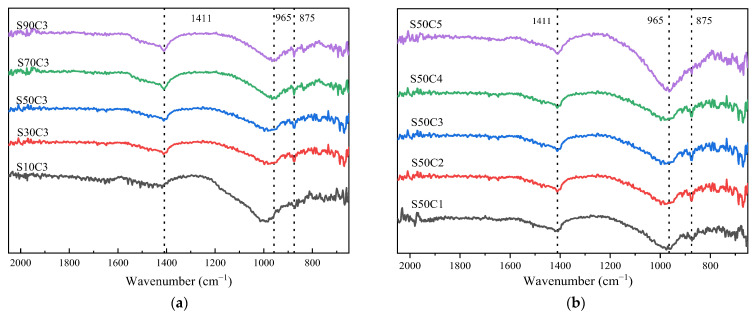
FT-IR spectra analysis of alkali-activated composite cementitious materials: (**a**) slag content; (**b**) activator concentration.

The effects of different combination ratios on the content of C-(A)-S-H gel in alkali-activated cementitious material were analyzed by XRD. However, N-A-S-H is an amorphous phase, which is difficult to be detected by XRD. Certain functional groups in N-A-S-H gel and C-(A)-S-H gel vibrate when exposed to continuously varying infrared light, corresponding to wave peaks in the FT-IR spectrum. Different types of functional groups can be distinguished according to the wave number corresponding to the wave crest. Therefore, N-A-S-H and C-(A)-S-H gels can be detected by FT-IR. The FT-IR spectra of alkali-activated fly ash–slag composite cementitious material at seven days of age at different mixing ratios are shown in Figure 6. The band at 875 cm^−1^ represents the vibration of the C-O-C bond in the carbonate [21]. The absorption band around 1411 cm^−1^ represents the stretching vibration of C-O bond in CO_3_^2−^ ion [38]. The band around 965 cm^−1^ is generated by the asymmetric telescopic vibration of the Si-O-T (T stands for Si or Al) dissolved by the [SiO_4_] tetrahedron and N-A-S-H gel, corresponding to the formation of C-(A)-S-H gels in the hydrated products [39].

Observing Figure 6, it was found that, although the same types of hydration products are present at different ratios, the difference between the hydration products of each group can be observed from the movement of the band. The energy required for vibration decreases when the peak moves to a low wavenumber. It can be seen from Figure 6 that the main peaks of Si-O-T bands of all alkali-activated cementitious materials are higher than those of other bands. As the slag content increases from 10% to 90%, the peak of the main peak of the Si-O-T band gradually shifts to a lower wavenumber, from 994 cm^−1^ to 965 cm^−1^. This is due to the large amount of Ca^2+^ dissolved in the slag into the N-A-S-H gel phase, partially replacing Na^+^. Moreover, with Al^3+^ replacing part of the Si^4+^, the original aluminum–silicon phase depolymerizes and participates in hydration, reflecting the transformation of N-A-S-H gel to C-(A)-S-H gel. Moreover, calcium in the non-crystalline structure of slag is easily dissolved in solution, and silicon or aluminum is more likely to form C-(A)-S-H gel with calcium. It can be concluded that, with the increase of slag content, more C-(A)-S-H gel is generated in the system, which is consistent with the XRD results. Through comparative analysis of the test data, it is found that, compared with slag, the activator concentration has less influence on the FT-IR test results. With the increase in the activator concentration from 0.20 to 0.40, the peak of the main peak of the Si-O-T band undergoes a weak migration, from 973 cm^−1^ to 964 cm^−1^. This is because the increase in the activator concentration stimulates the formation of Si-O-Si and Al-O-Si bonds in the system, forming an uncrystalline-state Si-O-T network structure. Furthermore, this improves the degree of polymerization of the cementitious material, promoting the formation of more N-A-S-H gels and converting them to C-(A)-S-H gels [7]. This indicates that the formation of C-(A)-S-H gel can be promoted by increasing the concentration of the activator appropriately, which is consistent with the XRD results. However, when the activator concentration reaches a certain value, the formation of cementitious material in the system is much faster than the aluminosilicate dissolution rate, and this polymerization reaction is inhibited.

### 3.5. Pore Structure Analysis

**Figure 7:**
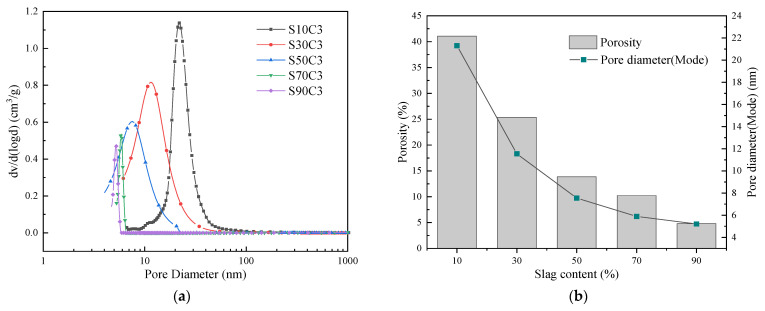
Results of the MIP analysis of alkali-activated composite cementitious materials under different slag contents: (**a**) pore size distribution; (**b**) porosity and the pore diameter (mode).

**Figure 8:**
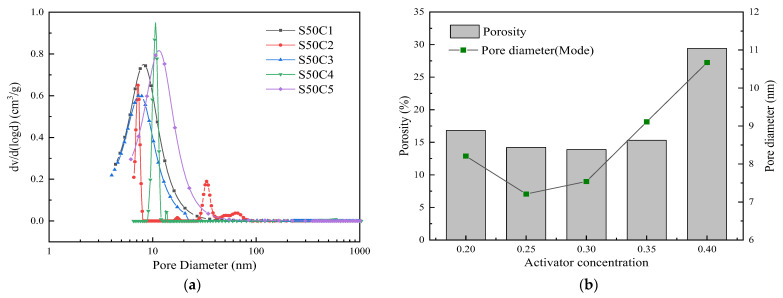
Results of MIP of alkali-activated composite cementitious materials under different activator concentrations: (**a**) pore size distribution; (**b**) porosity and the pore diameter (mode).

The pore size distribution and porosity of the structure have an important influence on the compressive strength of the net slurry. The results of different pore size distributions of MIP at seven days of age of alkali-activated fly ash–slag composite cementitious materials under different slag content and activator concentration are shown in Figure 7 and Figure 8.

It can be seen from Figure 7 that, when the slag content is 10%, the porosity reaches 41.08%, and the most probable pore size is 21.31 nm, which may be the main reason for the material’s low strength. With the increase in slag content from 10% to 90%, the peak point of the pore size distribution gradually shifts to the left, the most probable pore size decreases from 21.31 nm to 5.21 nm, and the porosity decreases from 41.08% to 4.81%. Moreover, the pore size of the alkali-activated fly ash–slag composite cementitious material becomes smaller and smaller, and the structure becomes more and more dense. This shows that the increase in slag is conducive to refining the pore size distribution of the system, reducing the size of its pores and greatly reducing the porosity. At the same time, when the slag content reaches 50% and above, the pore volume is mainly composed of gel pores (<10 nm). This indicates that, when the slag content reaches a certain value, the gel product inside the system increases, and the capillary pores are gradually filled by the gel product and converted into gel pores. As the volume of the gel pore increases, the pore size decreases and the structure becomes denser, thereby improving the strength of the alkali-activated cementitious material [40].

It can be seen from Figure 8 that the pore size distribution does not differ greatly under different activator concentrations, and the pore size is mainly distributed at about 10 nm. With the increase in the excitation concentration from 0.20 to 0.40, the porosity first decreases and then increases. At an activator concentration of 0.30, the porosity reaches a minimum value of 13.89%. When the activator concentration is 0.20, the most probable pore size is 8.21 nm, and the porosity is 16.83%. When the activator concentration is 0.25 and 0.30, the most probable pore sizes are 7.21 nm and 7.54 nm, and the porosity is 14.21% and 13.89%, respectively. The most probable pore size of 0.25 activator concentration is smaller than that of 0.30 activator concentration. However, it can be seen from Figure 8a that there is a pore size of about 30 nm in the 0.25 activator concentration, resulting in a porosity greater than 0.30 activator concentration and a compressive strength less than 0.30 activator concentration. When the concentration of the alkaline activator continues to increase to 0.40, the most probable pore size is 10.67 nm, the porosity is 29.41%, and both show an increasing trend. The above results show that, by adjusting the activator concentration, the amount of gel generated in the system can be increased and the proportion of gel pores can be increased. The pore size distribution of the structure can be refined, and the strength of alkali-activated cementitious materials can be improved [7]. However, compared with slag, the activator concentration has less of an influence on the pore structure of alkali-activated cementitious materials.

### 3.6. Microscopic Morphology Analysis

**Figure 9:**
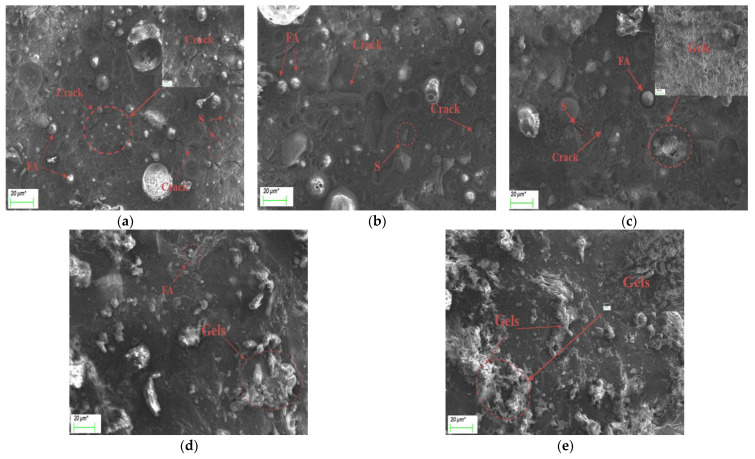
SEM images of alkali-activated composite cementitious materials under different slag contents: (**a**) S10C3; (**b**) S30C3; (**c**) S50C3; (**d**) S70C3; (**e**) S90C3. (μm* represents the unit μm).

**Figure 10:**
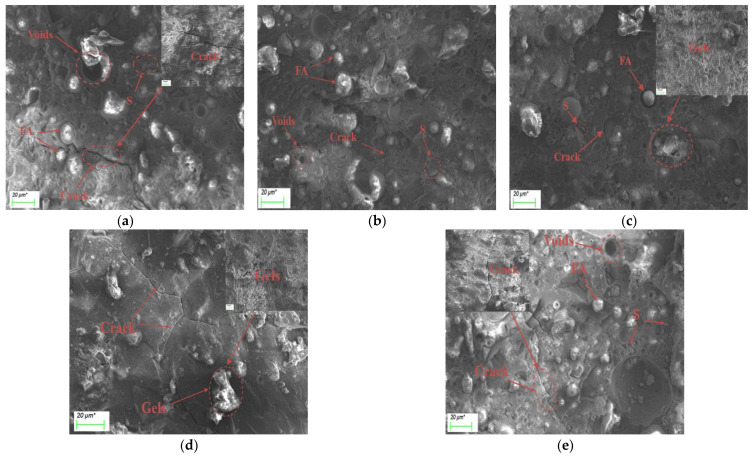
SEM images of alkali-activated composite cementitious materials under different activator concentrations: (**a**) S50C1; (**b**) S50C2; (**c**) S50C3; (**d**) S50C4; (**e**) S50C5. (μm* represents the unit μm).

The micromorphology of alkali-activated fly ash–slag composite cementitious materials under different slag content and activator concentrations at day 7 is shown in Figure 9 and Figure 10.

It can be seen in Figure 9 that the incorporation of slag has a significant impact on the morphology and structure of the alkali-activated fly ash–slag composite system. The higher the amount of slag, the denser the microstructure of the composite system, and the higher the strength of the cementitious material. When the slag content is 10%, it can be clearly seen that there are a large number of unreacted fly ash particles and large width cracks, and the structure is loose and porous. These phenomena confirm the results of the pore structure test in Section 3.5. When the slag content is small, the porosity of the structure is larger. The same phenomenon was also found in the study of Zhan et al. [41]. When the slag content was small, there were obviously unreacted fly ash particles and more cracks in the structure, and the hydration products showed an obvious massive structure. This is due to the low activity of fly ash and the need for curing in a high-temperature environment, with a curing temperature generally between 60 °C and 90 °C [42]. Therefore, fewer hydration products are generated, the structure is loose and porous and easily produces cracks, and the strength is low. When the slag content is increased to 50%, there are only a small number of unreacted fly ash particles and a low amount of unhydrated slag particles and microcracks. This is because the slag has higher activity, which improves the hydration reaction rate, generates a large amount of C-(A)-S-H and N-A-S-H gels, forms a denser and more uniform microstructure, and reduces the porosity. This is consistent with the results of the pore structure test. The increase of slag content can effectively reduce the porosity of the structure and thus improve the strength of the cementitious material [18]. When the slag content continues to increase to 90%, there are almost no unreacted fly ash and slag particles. A large amount of gel is generated, filling the pores and making the structure denser and more uniform than before, and the strength reaches its highest levels. The above results show that the incorporation of slag not only changes the composition of hydration products, but also promotes the dissolution and polymerization reaction of fly ash. A large number of C-(A)-S-H and N-A-S-H gels are generated, which reduces the porosity of the material and forms a denser cementitious structure, thereby improving the compressive strength of the material. Song et al. [40] pointed out that the increase of slag content can promote the generation of gel products, reduce the porosity of materials, ensure the formation of a denser gel structure, and thus improve the mechanical properties of materials.

It can be seen from Figure 10 that, when the activator concentration is 0.20, many unreacted fly ash particles, voids and wide cracks can be observed in the image. This is because the low alkaline environment provided is not conducive to the dissolution of minerals such as CaO, Al_2_O_3_, SiO_2_ in the alkali-activated materials, as it inhibits the hydration reaction and reduces the production of hydration products. As a result, the structure is loose and easy to produce cracks, and the porosity of the structure is large, which verifies the results of the pore structure test. When the activator concentration increases to 0.30, only a small number of unreacted fly ash particles were found in the image, the structure was relatively dense, and no obvious cracks are found. This is because, with the increase in the activator concentration, more Na_2_O and SiO_2_ are introduced into the system. This improves the alkaline environment of the material, makes the raw material particles dissolve more fully, accelerates the hydration rate, and generates more gels to fill the porous system. The above phenomenon is consistent with the results of the pore structure test. Appropriately increasing the activator concentration can effectively reduce the porosity of the structure, make the structure more compact and uniform, and significantly improve the strength [29]. When the activator concentration continues to increase to 0.40, there are unreacted fly ash and slag particles, cracks, and holes in the composite architecture, resulting in a decrease in strength. This is because the excessive activator concentration makes the early hydration reaction occur too quickly. The resulting film of hydration products envelops the undissolved silicon–aluminum phase, slowing down the subsequent hydration reaction and resulting in reduced strength [21].

## 4. Conclusions

Taking the slag content and activator concentration as the experimental variables, the influence of these two factors on the compressive strength of alkali-activated fly ash–slag composite cementitious materials under different curing ages was studied. Using micro-technical means, the intrinsic influence mechanism of the slag content and activator concentration on the material’s mechanical properties was explored. The main conclusions are listed below:

- The compressive strength of alkali-activated cementitious materials increases as the curing age increases. The increased curing age increases the degree of hydration reaction of alkali-activated cementitious materials, and more hydration products are generated, thereby improving the compressive strength. Moreover, the hydration reaction is relatively rapid in the early stage and is relatively slow in the later stage, showing the characteristics of early strength.
- The amount of slag is the main influencing factor in the compressive strength of alkali-activated cementitious materials, and the compressive strength of alkali-activated cementitious materials tends to increase with an increase in slag content, showing higher mechanical properties. Compared with slag, the activator concentration has less of an effect on the compressive strength of alkali-activated cementitious materials. As the activator concentration increases from 0.20 to 0.40, it first increases and then decreases, and the compressive strength reaches its maximum at 0.30.
- Based on the analysis of the results of the microstructure test, it was found that the increase in slag content accelerates the hydration reaction rate of alkali-activated cementitious materials. It promotes the formation of a large number of C-(A)-S-H and N-A-S-H gels, refines the pore size distribution, and forms a denser and more uniform microstructure, thereby improving the strength of the cementitious material. The increase in the activator concentration improves the alkalinity of the activator. This promotes the production of more gel products and increases the strength of the cementitious material. When the activator concentration increases to a certain value, the strength of the alkali-activated cementitious material begins to decrease; when the activator concentration is too large or too small, this will hinder the hydration reaction and affect the development of strength.

## Figures and Tables

**Table 1:** Chemical composition of fly ash and slag.

Material	Mass Fraction/%								
	CaO	SiO_2_	Al_2_O_3_	Fe_2_O_3_	MgO	SO_3_	K_2_O	TiO_2_	Others
Fly ash	2.66	55.71	32.80	4.43	0.23	0.65	1.54	1.66	0.32
Slag	44.06	30.23	13.72	0.41	5.58	3.16	0.50	1.79	0.55

**Table 2:** Mixed-ratio alkali-activated fly ash–slag composite cementitious material.

Group	Sample No.	Alkali-Activated Materials		Activator Concentration	Water-to-Binder Ratio	Modulus
		Fly Ash/%	Slag/%			
Slag content group	S10C3	90	10	0.30	0.35	1.2
	S30C3	70	30			
	S50C3	50	50			
	S70C3	30	70			
	S90C3	10	90			
Activator concentration group	S50C1	50	50	0.20	0.35	1.2
	S50C2			0.25		
	S50C3			0.30		
	S50C4			0.35		
	S50C5			0.40		

## Data Availability

Data is contained within the article.
